# Testosterone treatment reveals marked sex differences in song diversity and syllable syntax in adult canaries

**DOI:** 10.21203/rs.3.rs-2755085/v1

**Published:** 2023-04-11

**Authors:** Ednei B. dos Santos, Gregory F. Ball, David M Logue, Charlotte A Cornil, Jacques Balthazart

**Affiliations:** University of Liege: Universite de Liege; University of Maryland at College Park; University of Lethbridge Department of Psychology; University of Liege: Universite de Liege; University of Liege: Universite de Liege

**Keywords:** song diversity, syllable sequences, sex differences, testosterone, network analysis

## Abstract

**Background.:**

Behavioral sex differences are widespread in the animal world. These differences can be qualitative (i.e., behavior present in one sex but not the other, a true sex dimorphism) or quantitative (behavior is present at a higher rate or quality in one sex compared to the other). Singing in oscine songbirds is associated with both types of differences. In canaries, female rarely sing spontaneously but they can be induced to do so by treatments with steroids. Song in these females is however not fully masculinized and exhibits relatively subtle differences in quality as compared with male song. We analyzed here sex differences in syllable content and syllable use between singing male and female canaries.

**Methods.:**

Songs were recorded from 3 groups of castrated male and 3 groups of photoregressed female canaries that had received Silastic^™^ implants filled with testosterone (T), with T plus estradiol (E2), or left empty (control). After 6 weeks of hormone treatment, 30 songs were recorded from each of the 47 subjects. Songs were segmented and each syllable was annotated. Various metrics of syllable diversity were extracted and network analysis was employed to characterize syllable sequences.

**Results.:**

Male and female songs were characterized by marked sex differences related to syllable use. Compared to females, males had a larger syllable type repertoire and their songs contained more syllable types. Network analysis of syllable sequences showed that males follow more fixed patterns of syllable transitions than females. Both sexes however produced song of the same duration containing the same number of syllables produced at similar rates (numbers per second).

**Conclusions.:**

Under the influence of T canaries of both sexes are able to produce generally similar vocalizations that nevertheless differ in specific ways. The development of song during ontogeny appears to be a very sophisticated process that is presumably based on genetic and endocrine mechanisms but also on specific learning processes. These data highlight the importance of detailed behavioral analyses in order to identify the many dimensions of a behavior that can differ between males and females.

## Summary

Male canaries normally sing complex songs at high rate while females only rarely sing very simple songs. Testosterone induces active singing in both male and female canaries but female song is still not fully masculinized by these treatments even if song duration does not differ between the sexes. We analyzed the syllable repertoire and the sequence of use for different syllables in canaries of both sexes treated with testosterone or testosterone supplemented with estradiol. Compared to females, males had a larger syllable type repertoire and their songs contained more syllable types. Syllable transitions were also more fixed in males. Sex differences in adult singing of canaries are thus a complex mixture of differences that only result from the different endocrine condition of males and females (and are thus partially reversed by administration of exogenous testosterone) and of more stable differences that presumably develop during the ontogenetic process under the influence of endocrine and genetic differences and of differential learning processes. Canary song thus represents an outstanding model system to analyze the interaction between nature and nurture in the acquisition of a sophisticated learned behavior as well as sex-typical patterns of vocal learning and production.

## Introduction

Birdsong and the supporting network of brain nuclei, the song control system, have emerged as a widely used model system to analyse biological mechanisms underlying important phenomenon including vocal learning, brain plasticity, brain lateralization, steroid action on brain and behavior, and also sex differences in brain and behavior (reviewed in [1–7]). In many songbird species including canaries, syllable types are the building blocks for songs, with song diversity arising from the flexibility with which different syllable types are combined in song construction. These syntax-like combinatorial properties have often been compared to human language (reviewed in [2, 3])

Different aspects of song and syllable diversity have been shown to predict breeding success and pairing date in songbird species such as sedge warblers *Acrocephalus schoenobaenus* [8], eastern song sparrow *Melospiza melodia melodia* [9] and house wrens *Troglodytes aedon* [10]. Complex singing patterns are hypothesized to function as signals for male quality assessment by rivals and/or potential mates [11, 12]. Females that choose males that sing more diverse (or complex) songs may mate with higher quality males and obtain greater direct benefits from them, such as better parental care and a larger territory with more resources, or greater indirect benefits in the form of superior genes that improve offspring in various ways including enhanced immunocompetence [13, 14]. In turn, males that sing more diverse songs might acquire extra-pair fertilizations, multiple mates, or mates of superior quality and have increased reproductive success as a result. In canaries (*Serinus canaria*) specifically, Kroodsma showed in a classic study [15] that females build nests faster and lay more eggs when exposed to playbacks of male songs with higher syllable diversity.

We also know that male songs containing special syllables that females prefer induce a higher rate of immediate early gene expression in females as compared to male song without these syllables [16]. Additionally, songs produced by T-treated female canaries were recently shown to be able to enhance immediate early gene expression in the auditory forebrain of other females, but it is not known if this expression is equivalent to what happens in response to male song [17]. It is also important to remember that, although there are correlations between aspects of song that we can measure and measures of female fitness, studies of song perception in songbirds suggest that aspects of song that seem important to us such as syllable sequence are not easily discriminable in certain species such as zebra finches [18].

Studies on birdsong are historically male-biased, partly because research has focused on northern temperate zone species, in which males sing more often than females. However, recent studies have shown that song in both sexes is probably the ancestral condition in songbirds (suborder Passeri) and that female song is much more common than previously thought [19, 20].

Female song has therefore been largely understudied and in particular, studies reporting quantitative information about sex differences in song and syllable type usage are rare, presumably due, at least in part, to the fact that cataloguing and annotation of syllable type repertoires are much more time-consuming than scoring time (rate, duration) and frequency domain of song variables. In the present study we address this gap in the literature by providing a thorough analysis of song and syllable diversity in male and female canaries.

Both male and female canaries sing but spontaneous female song is both more plastic and less common than male song. A recent study characterizing spontaneous female song in canaries estimated that less than 7% of females raised in aviary conditions sing [21]. However, female canaries can be induced to sing by treatments with exogenous steroid hormones and are, as a consequence, commonly used in experiments assessing the effects of sex steroids on singing behavior and brain anatomy [22, 23]. After 2 to 3 weeks of testosterone (T) treatment, females start singing at high rates [24, 25]. Previously, we reported that several measures of singing behavior and song structure are activated more efficiently both by T and T plus estradiol (E2) treatments in male than in female canaries. These steroid treatments also increase the volume of forebrain song control nuclei (HVC, RA, and Area X) in both sexes, but these nuclei remain significantly smaller in females than in males irrespective of treatment [25]. Ongoing additional analyses of results from this experiment also suggested that sex differences in trill production are related to sex differences in syrinx mass. Males produce songs with more trills and have heavier syringes and these differences cannot be reversed by sex steroids in adulthood (Dos Santos et al. unpublished data).

Here, we examined potential sex differences in syllable diversity and use in male and female Fife fancy canaries that had been treated with sex steroid hormones. In addition to the song and syllable diversity analysis, we employed a set of network analysis tools to visualize and quantify variability in syllable type sequences [26–29].

## Material And Methods

### Study subjects

The present analyses concern recordings that were collected in a published experiment. Experimental procedures were previously described in detail in a published paper [25] and are just briefly summarized here.

One-year old Fife Fancy canaries (23 females and 24 males) were acquired from a commercial breeder in Antwerp, Belgium. We housed birds indoors in wire cages (49 × 95 × 51 cm) at the University of Liege, Belgium. Males and females were kept in separate cages in groups of 6 individuals on an 8 L:16 D (light/dark) cycle that was maintained throughout the experiment. They received seed mix and water *ad libitum*, as well as baths and grit daily, and eggfood (blended hard-boiled chicken eggs and bread) twice a week. The sex of birds was determined via molecular sexing at the Behavioral Ecology and Ecophysiology lab animal facility of the University of Antwerp, Belgium [27].

### Surgical procedures

Six weeks after arriving at our animal facilities, males were castrated under general anesthesia induced by a mix of air gas and 3% isoflurane for induction and 2.5% for maintenance. Testes were regressed at the time of castration, which indicates that males were in non-breeding condition. Testes were removed through a small unilateral incision in the left flank, between the last two ribs. After suturing the incision, we allowed males to recover under a warm light before returning them to their cages. Females were not ovariectomized but were laparotomized to confirm that ovaries were photoregressed. The high level of vascularization of the ovary in female canaries makes it difficult to perform ovariectomies without inducing a high rate of mortality. Previous studies have successfully used intact females that had photoregressed ovaries, so our approach facilitates comparison with previous work [24, 25, 30, 31]. Females were similarly returned to their home cages after they had recovered from surgery under a warm lamp.

### Hormone Implants

Three weeks after surgeries, subjects received subcutaneous implants made with Silastic ^™^ tubing (Dow corning, Midland, MI, USA; Degania Silicone; internal diameter 0.76 mm, external diameter 1.65 mm, length 10 mm) filled with either crystalline steroids or left empty as a control. Implants were pre-incubated in 0.9% saline at 37°C overnight and inserted subcutaneously through a small hole in the back of the birds. Subjects were kept under isoflurane anesthesia during the surgical procedure and the hole was sutured with a thread (5 − 0 coated Vicryl^™^). Birds (n = 8 of each sex per experimental group) were implanted with Silastic^™^ implants filled with either T or T + E2, or left empty (control). The T + E2 group was included because previous studies reported that the induction of aromatase activity by T is lower in the female than in the male brain in all mammalian and avian species that have been studied (see [25] for references). This sex difference in aromatase activity might explain the limited behavioral response of females to exogenous T [24, 25] since activation of several features of song is mediated by estrogens derived from T aromatization [32–35]. Therefore, we added exogenous E2 in an effort to compensate for this possible sex difference.

### Song recordings and annotations

Six weeks after the onset of steroid treatment each bird was recorded for 3 h starting immediately after the lights went on (0900 h). Individual birds were placed on the previous day inside custom-built sound-attenuated boxes. Songs were recorded using custom-made microphones (Projects Unlimited/Audio Products Division) and an Allen & Heath ICE-16 multichannel recorder. The sound files were acquired and saved as .wav files by Raven v1.4 software (Bioacoustics Research Program 2011; Raven Pro: Interactive Sound Analysis Software, Version 1.4, Ithaca, NY: The Cornell Lab of Ornithology) at a sampling frequency of 44,100 Hz which translates to a frequency range of 0–22,050 Hz.

We analysed the first 30 songs produced by each individual. Vocalizations were defined as songs if they were at least 1 s long and were preceded and followed by at least 0.4 s of silence as done in our previous work [25]. We defined syllables as grouped combinations of sounds that are consistently produced together as a common unit. We defined syllable types based on their appearance on a sound spectrogram in order to develop a catalog of syllables for each individual. Songs were then segmented and their syllable content annotated using PRAAT, version 6.2.23 (see https://www.fon.hum.uva.nl/praat/).

### Diversity metrics

We measured eight variables that are conventionally used to capture song diversity (or complexity) at multiple levels [10, 36]: *song repertoire size* (number of different song types), *syllable type repertoire size* (number of different syllable types), *number of syllables per song, number of syllable types per song, number of syllable types per second, number of syllables per second, syllable versatility index* (SVI; number of syllable types/number of syllables) and *Levenshtein distance*. Levenshtein distance measures variation in syllable type sequences between successive songs. It does this by quantifying the minimum number of edits required to convert a string of characters, such as a sequence of syllables, into another using single-place deletions, insertions or replacements of individual characters [37].

### Network analysis of syllable type sequences

Based on graph theory, network analysis has been applied to a wide range of scientific disciplines, from molecular networks to social organization. In animal communication, network analysis tools have been used as a complement to more traditional measures of song diversity to assess properties of song organization and diversity that are not revealed by other approaches [29], including whether patterns of syllable transitions are more linear and fixed or more variable.

Thus, in addition to the diversity metrics listed above, we analysed the sequences of syllable types using a network-based approach [26, 27, 29]. Syllable type sequence networks were created for each individual from a string of successive syllable types over a sample of 30 songs (the same used for the previous set of analyses). The breaks between songs were ignored to avoid creating small, disconnected networks [29]. Networks comprised of three or fewer syllable types were removed from further analysis to avoid bias resulting from the limited number of nodes [29].

Syllable type networks were created in R (R Core Team 2022) using the igraph package [38]. Calculations were based on undirected networks. We assigned syllable types as the nodes and the first-order transitions between syllable types as the edges of the network. In order to quantify transition relationships between syllable types, we used two network variables: the *average minimum path length (path length in short)* and the *network density*. These metrics allowed us to characterize song structure by the organization of its basic components and measure flexibility of syllable type transitions [29].

*Average path length* is the average of the minimum number of edges (transitions) required to link any pairs of nodes (syllable types) in a network while ignoring dead ends. Higher values of average path length indicate that more transitions are required to connect any given pair of syllable types. This suggests that syllable sequences are produced in a more constrained or fixed fashion; different syllables are produced in relatively fixed sequences and there is less connectivity between syllable types. Conversely, shorter average path lengths indicate less regularity in syllable type sequences. In other words there is more flexibility in the transitions between different song types

Another measure of networks reported here is the *Network* density which is computed as the ratio of the number of edges observed in the network divided by the number of all possible syllable transitions in the network given its size. Here higher values indicate that more transitions do occur so that syllable sequences are more flexible while lower values indicate more limited transitions and thus more stereotypy (see [29] for more detail).

### Statistical analysis

Unless otherwise mentioned, data were analyzed by one- or two-way analyses of variance (ANOVA), or by two-way mixed model analysis, if a few data points were missing. The 3 experimental groups and 2 sexes were considered as independent factors. Statistical analyses were performed using GraphPad Prism version 8.2.1 for Mac (GraphPad Software, San Diego, California USA). We used an alpha level of 0.05 for all statistical tests. All data are represented here by their mean ± SEM.

## Results

The total sample comprised 47 individuals (24 males and 23 females). Songs from control birds were excluded from the analysis because they are mostly characteristic of the plastic phase of development when syllables that are produced are not yet stereotyped [39, 40]. Hence, it was not possible to clearly delineate their syllable content. The final sample comprised a total of 840 songs (57,872 syllables) from 15 males and 13 females from the T and T + E2 groups. (see Table S1 for details).

### Male songs show higher diversity

**Figure 1:**
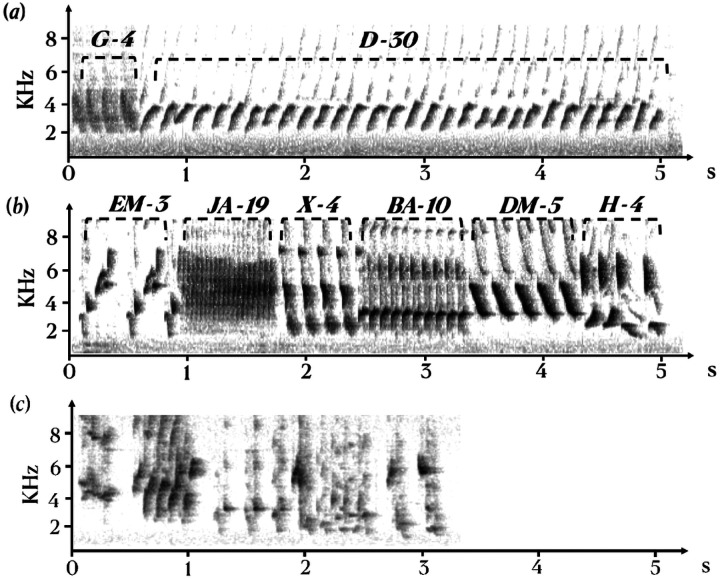
Spectrograms of canary songs after 6 weeks of treatment with steroids. *(a) female, (b) male*, (c) typical plastic song from control male with unclear syllable shapes that could not be classified. *The different syllable types were catalogued and labeled based on clear and consistent differences in overall temporal-spectral shape that were repeatable across songs both within and between birds*. The labels of the different syllable types are indicated above the sonograms.

Spectrograms indicated the presence of stereotyped repeatable syllables in the songs of males and females treated with T or T and E2 but not in control birds (Fig. 1).

No qualitative difference could be detected between the two steroid-treated groups (T vs. T + E2) within each sex.

**Figure 2:**
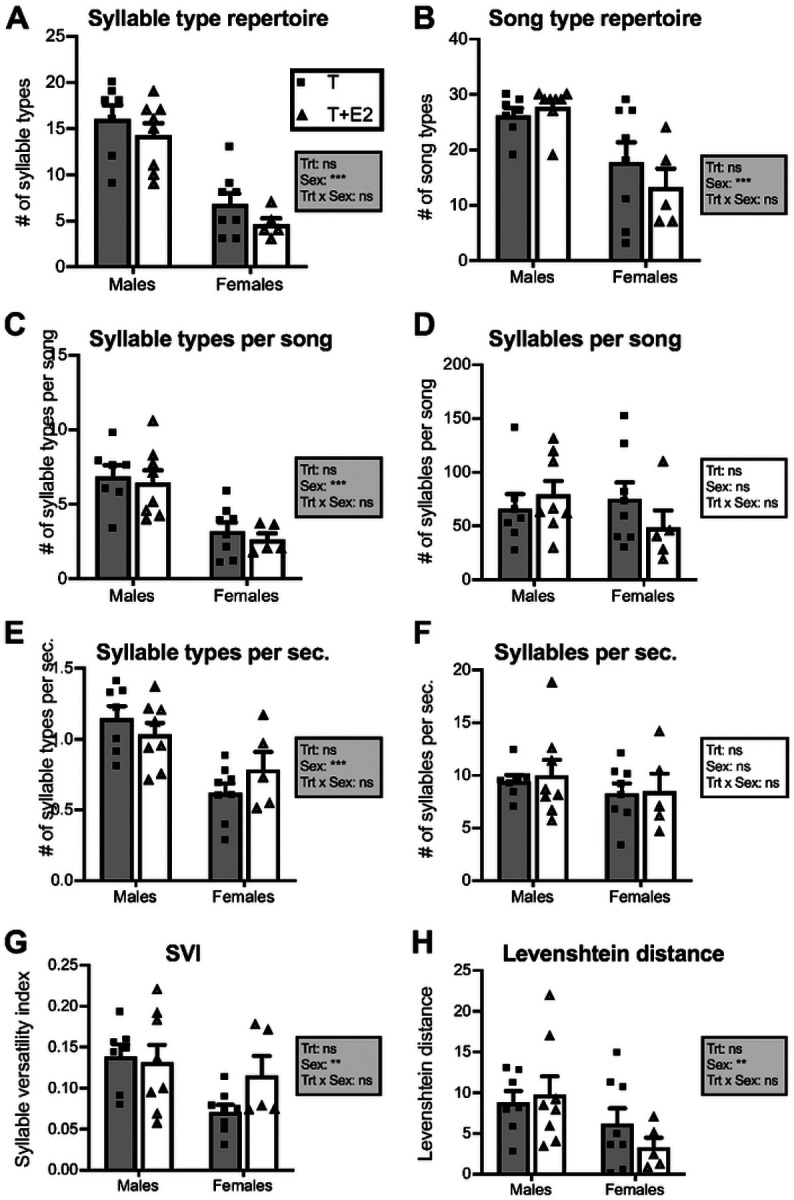
Song and syllable diversity metrics in male and female canaries that were treated with Silastic^™^ implants filled with testosterone (T; gray bars) or with testosterone plus estradiol (T+E2; white bars). Bar graphs represent the mean ± SEM of results that are the average of the first 30 songs produced by each experimental individual after six weeks of steroids treatment. Individual data points are also indicated. Data were analyzed by two-way ANOVA with treatment (Trt) and Sex of the subjects as independent factors and results are summarized in the insert for each panel. (***=p<0.001, **=p<0.01, *=p<0.05, ns=not significant). See Table 1 for the detail of statistical results..

Quantitative analyses of 8 variables characterizing these songs by two-way ANOVAs (treatment and sex as independent variables) identified no overall effects of the treatments by steroids (T vs. T + E2) and no significant interaction between the two factors (See Fig. 2 and Table 1). In contrast, these ANOVAs detected significant sex differences for six variables: syllable type repertoire (Fig. 2A), song type repertoire (Fig. 2B), numbers of syllable types per song (Fig. 2C), and numbers of syllable types per second (Fig. 2E) were significantly larger in males than in females. This was also the case for the Syllable Versatility Index, SVI (Fig. 2G) and Levenshtein distance (Fig. 2H). However, ANOVAs did not reveal significant sex differences in the number of syllables per song (Fig. 2D) or the number of syllables produced per second (Fig. 2F).

Female thus sang songs of the same overall length as males (as previously reported in [25]) that contained the same number of syllables produced at the same rate (numbers per second) but these female songs contained many repeats of the same syllable types, as clearly visible in Fig. 1

### Network analysis: Male songs show higher linearity in syllable type transitions

**Figure 3:**
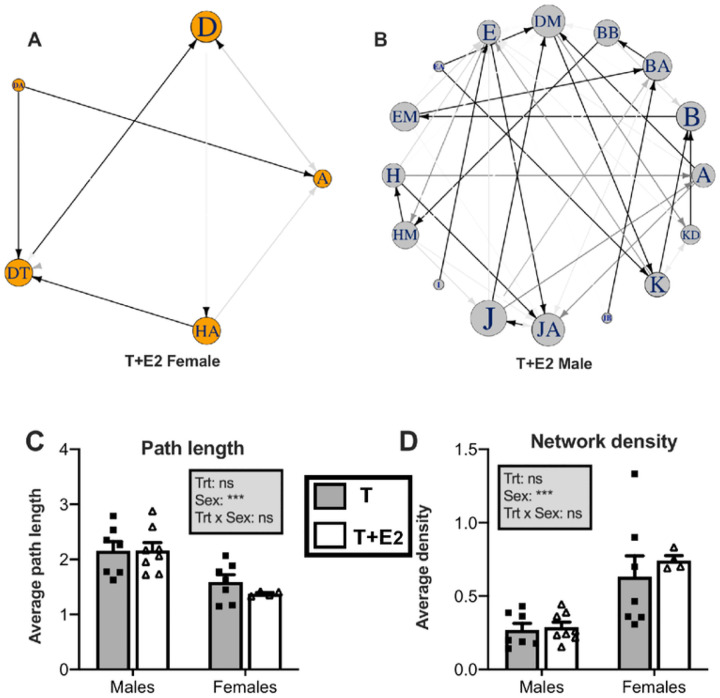
Examples of diagrams illustrating difference in syllable type use in female (A) and male (B) canaries treated with sex steroids. Each panel is constructed from a string of successive syllable types over a sample of 30 songs, and the first-order transitions between them. Each circle represents a different syllable type, and differences in the size of the circles capture variation in their relative use. Lines between circles represent syllable type switching, with the arrow indicating the direction of the transition and the darkness of the line is proportional to the relative frequency of that particular transition. **C – D** illustrate the quantitative sex differences in syllable type use. Bar graphs represent the mean ± SEM of results. Individual data points are also indicated. Data were analyzed by two-way ANOVA with treatment (Trt) and Sex of the subjects as independent factors and results are summarized in the insert for each panel. (***=p<0.001, **=p<0.01, *=p<0.05, ns=not significant).

Representative examples of networks computed for a female and a male of the T + E2 group are presented in Fig. 3B and B. There were significant sex differences for all both network metrics that were used to characterize linearity in syllable type transitions: overall network path length was higher in males than in females (F_1, 24_ = 25.38, *p* < 0.0001, Fig. 3C) while the opposite was seen for network density (F_1, 24_ = 40.37, *p* < 0.0001, Fig. 3D). There were no significant treatments or interaction effects (all p > 0.33)

## Discussion

Here we provide a detailed quantitative characterization of song structure and organization for male and female canaries treated with sex steroids. These analyses identified dramatic sex differences in song and syllable type diversity, and in syllable type sequences. Overall, males sang songs that contain a higher number of syllable types when compared to females. In addition, male songs follow more fixed patterns of syllable type transitions than female songs. Taken together, these results agree with our previous reports showing that different measures of singing behavior and song structure (in the frequency and time domains) [25], and trill production (Dos Santos et al. unpublished data) are activated more efficiently both by T and T plus E2 treatments in male than in female canaries. The present data also confirm that supplementing T with exogenous E2 does not enhance the responses of females to T.

### Male songs are more diverse at multiple levels

Both males and females concatenated syllable types into songs in similar ways, often showing patterns of trill production (serial repetition of the same syllable type) but male songs contained a much higher number of different syllable types than female songs (Fig. 2C). The present analyses thoroughly captured song and syllable diversity at multiple organizational levels. First, at a global level, males exhibited larger song type (Fig. 2B) and syllable type (Fig. 2A) repertoires than females. There were also significant sex differences in metrics that captured within songs variability, such as the *number of syllable types per song* (Fig. 2C), *number of syllable types per second* (Fig. 2E), and *syllable versatility index* (Fig. 2G). Finally, we detected significant sex differences in *Levenshtein distance*, a variable that captures variation between successive songs in the sequence of specific syllable types they contain [36, 37]. Overall, females thus produced songs that are very stereotyped with many repeats of the same syllable types. It is important to note that most measures were affected by a substantial degree of individual variability and some females showed a greater diversity in their singing behavior, at times producing a number of song and syllable types closer to typical male songs [41]. Vallet and collaborators also pointed out these individual differences among testosterone treated females, observing that their repertoire size shows large individual differences.

Interestingly, males and females do not differ in syllable production as measured by the number of *syllables per song* (Fig. 2D) and number of *syllables per sec*. (Fig. 2F). This is in line with a previous report showing no sex differences in the number of syllables per song and time vocalizing within songs (Fig. 2 in [25]), although we showed that males produce more songs and trills overall. Hence, sex differences seem to be present at the global level of song production, but also in a more subtle manner within songs where they are restricted to the diversity and organization and syllable use.

### Male songs show higher stereotypy in syllable type transitions

We also found sex differences in patterns of transitions between syllable types. The network analysis of syllable sequences show that male songs follow more linear patterns of syllable type transitions than female songs. We detected very significant sex differences in the two network variables used to characterize syllable type transitions. Male songs showed significant higher values of *path length* than female songs (Fig. 3C). Thus, in male songs more transitions are required to connect any given pair of syllable types, suggesting that syllable type sequences are produced in a more regular and fixed fashion. In contrast, females showed significant higher values of *network density* (Fig. 3D). Thus female songs use a higher percentage of the possible transitions between syllable types in a given network and thus display relatively more flexibility in syllable sequencing. Together, these results are reflecting song sequences that are less linear and more flexible in the transitions among syllable types in females as compared to males.

### Function

Some female canaries actually sing spontaneously without addition of exogenous T but this only concerns a small proportion of the subjects (6 out of 112 in [21]). The spontaneously produced songs are however shorter than male songs or songs produced by females after treatment with testosterone. So T is able to enhance and masculinize some of the features of song in females (e.g., song duration, number of syllables per song) but some other features remain markedly different (e.g., number of syllable types used and their syntax).

Given what is known about sexual partner female choice in songbirds (see introduction), it is clear that the more complex songs of males should be preferred over songs that females can produce even in response to exogenous T. The variables that become identical in males and females have presumably not been submitted to an intense selection pressure during evolution while those features that remain sexually differentiated presumably have a high fitness value for males and have probably been actively selected. Note however that this inference is based on songs produced by T-treated females that obviously do not exist in nature. This idea must therefore be considered with caution.

The exact features of song that birds attend to when engaging in social behaviors including mate choice are difficult to discern. In zebra finches one may think that the sequence of syllables is important to females but when one conducts perceptual studies zebra finches have trouble identifying changes in syllable sequence while they can discriminate changes at the individual syllable level quite easily [42, 43]. This is in contrast to budgerigars that seem to discriminate variation in syllable sequence quite well [43]. Canaries on the other hand can perceive small variation in syllable structure (a syllable played backward is readily identified) but their overall approach to perception seems to be at the level of a phrase. Overall, these findings challenge the assumption that syllable sequence is significant for at least some songbird species in mediating social interactions but that other macro and micro features of song are clearly significant [18].

### Ontogeny

One can wonder how these stable sex differences affecting in a very specific manner some aspects of singing behavior, but not others, develop during ontogeny. It has been demonstrated that female canaries that received the same treatment with exogenous T as males still possess smaller song control nuclei (HVC, RA and Area X; [24, 25]) and exhibit major sex differences in gene expression in HVC [44] and RA [45]. Transcriptomes are also very different between spontaneously singing females and females signing after T treatment [44]. Thus although adult T treatment drastically alters gene expression, many genes remain expressed in a different way with parts of the transcriptome being male-biased while other parts are female-biased. This study thus indicates that singing behavior relies on the expression of different genes in male and female canaries. Some of the male-biased genes are located on the Z chromosome, consistent with previous work demonstrating that dosage compensation is limited in birds (males have 2 Z chromosome while ZW females only have one; [46–48]. However, the majority of sex-biased genes are located on autosomes. Their differential expression is thus presumably resulting from a secondary regulation by transcription factors or induced via epigenetic mechanisms by different life experiences that might include a differential early exposure to sex steroids as well as different interactions with their conspecifics.

It is well established that some aspects of the reproductive behavior of both birds and mammals are irreversibly differentiated following exposure to sex steroids during an early stage of development. In Japanese quail, exposure of females to their ovarian estrogens before day 12 of incubation irreversibly demasculinizes copulatory behavior. This effect can be mimicked in males by an injection of exogenous estradiol benzoate in the egg and blocked in females by an injection of an aromatase inhibitor that will prevent endogenous estrogen production [7]. The same mechanism appears to control the differentiation of copulatory behaviour in zebra finches [49] but the role of steroids in the control of the differentiation of the song system and of singing behaviour in this species remains unclear at present [7]. It is likely that sex steroids are implicated in this sexual differentiation [50, 51] but how exactly is unknown (for review see [7]).

Based on studies of a gynandromorphic zebra finch (half male-half female), it has also been demonstrated that genes affect sex differences of the song system independent of the differentiating effects of gonadal steroids [52]. Several genes that might be implicated in these “direct” effect have even been suggested see [7] for references) but the relative contribution of sex steroids and of “direct” genetic effects is not yet fully understood.

How these data apply to other songbird species, such as canaries, that show a less extreme sexual differentiation [53] is also not known but based on the repeated demonstration that some aspects of song cannot be fully masculinized by adult treatments with sex steroids, it appears very likely that these aspects are sexually differentiated in the organizational sense by the early action of sex steroids or genes. This idea should now be experimentally tested via hormonal or genetic manipulation of young nestlings.

Finally, it is likely that, during ontogeny, females do not learn the same amount as males about conspecific song. Song learning is known to occur in female songbirds in order to permit male song recognition [54, 55] but females obviously do not practice these learned vocalizations as much as males do during the period of sensorimotor learning (plastic song). Therefore they probably do not consolidate this memory to the same extent as males. In addition, it has been demonstrated that in zebra finches, the interaction of the young nestling with its tutor plays a critical role in song learning [56]. Male and female nestlings can thus live in the same environment but be exposed to a different *Umwelt* [57]. This differential earning probably contributes to explain the more limited syllable repertoire of T-treated females as compared to males so that under the influence of T these females end up producing songs of the same length as males but they are unable to populate them with a variety of diverse syllables.

Acquisition of song in female, and as a consequence male, canaries thus appears to be a very sophisticated process that is presumably based on genetic and endocrine mechanisms but also on specific learning processes during ontogeny. As such this represents an outstanding model to analyze the interaction between nature and nurture in the acquisition of a sophisticated learned behavior.

### Perspectives and Significance

This study demonstrates that, even if female canaries can be induced to sing at rates by a treatment with exogenous testosterone, some aspects of the song they produce, in particular the syllable repertoire and syllables organization into phrases, remain significantly different. The song of canaries is thus composed of aspects that are different between males and females only because the adults are exposed to different concentrations of hormones but it also contains aspects that cannot be masculinized by adult hormones and are thus probably sexually differentiated in the organizational sense. Future work should be devoted to the analysis of these organized aspects, in a first step by manipulating the neonate and juvenile endocrine environment, possibly in association with the exposure to an enriched acoustic environment.

## Figures and Tables

**Table 1:** Results of the two way ANOVAs comparing multiple song metrics in males and females treated for 6 weeks with T or T + E2.**Treatment Sex Interaction**

Variable	F _df_	P	F _df_	P	F _df_	P
Syllable type repertoire	F_1,24_ = 2.510	0.155	F_1,24_ = 50.53	0.0001***	F_1,24_ = 0.226	0.882
Song type repertoire	F_1,24_ = 0.306	0.585	F_1,24_ = 18.62	0.0002***	F_1,24_ = 1.341	0.258
Syllables/song	F_1,24_ = 0.199	0.659	F_1,24_ = 0.526	0.475	F_1,24_ = 1.782	0.194
Syllable types/song	F_1,24_ = 0.452	0.508	F_1,24_ = 26.20	0.0001***	F_1,24_ = 0.008	0.928
SVI	F_1,24_ = 1.085	0.308	F_1,24_ = 5.532	0.0272*	F_1,24_ = 2.076	0.162
Syllables/sec	F_1,24_ = 0.091	0.7660	F_1,24_ = 1.127	0.299	F_1,24_ = 0.021	0.886
Syllable types/sec	F_1,24_ = 0.091	0.766	F_1,24_ = 18.66	0.0002***	F_1,24_ = 2.420	0.133
Levenshtein distance	F_1,24_ = 0.250	0.622	F_1,24_ = 5.362	0.0294*	F_1,24_ = 0.968	0.335
Network path	F_1,22_ = 0.465	0.502	F_1,22_ = 20.07	0.0002***	F_1,22_ = 0.520	0.478
Network density	F_1,22_ = 0.557	0.463	F_1,22_ = 21.90	0.0001***	F_1,22_ = 0.281	0.602

The data reports the F ratio, degrees of freedom and associated probabilities for the 2 main factors (treatment i.e., T vs. T + E2 and sex) as well as their interaction.

## Data Availability

The dataset(s) supporting the conclusions of this article is(are) included within the article and one additional Excel file located at the end of this manuscript (Table S1 Dataset EBdS).

## Highlights

- Testosterone activates active singing in male and female canaries
- Male songs contain a larger syllable repertoire compared to females
- Syllable sequences in male songs are produced with more fixed transitions and less flexibility
